# Complicated orolingual angioedema after recombinant tissue plasminogen activator treatment in stroke patients under angiotensin converting enzyme inhibitor: Report of two cases

**Published:** 2015-10-07

**Authors:** Mohammad Reza Motamed, Fereshteh Nasiri, Seyed Mohammad Fereshtehnejad, Masoud Mehrpour, Babak Zamani, Bahram Haghi-Ashtiani, Farzaneh Rohani

**Affiliations:** 1 Department of Neurology, School of Medicine, Iran University of Medical Sciences, Tehran, Iran; 2 Care Sciences, and Society (NVS), Karolinska Institute AND Department of Neurobiology, Stockholm, Sweden AND Firoozgar Clinical Research Development Center, Firoozgar Hospital, Iran University of Medical Sciences; Tehran, Iran; 3 Firoozgar Clinical Research Development Center AND Department of Neurology, School of Medicine, Firoozgar Hospital, Iran University of Medical Sciences; Tehran, Iran

**Keywords:** Orolingual Angioedema, Stroke, Angiotensin-Converting Enzyme Inhibitors, Recombinant Tissue Plasminogen Activator

Currently, the intravenous (IV) recombinant tissue plasminogen activator (rt-PA) is approved as a relatively safe treatment approach to improve the outcomes following the acute ischemic stroke.^        1 ^ As an uncommon complication, angioedema occurred in 1.3-5% of stroke patients after IV rt-PA;^        2 ^ however, previous reports warn that patients taking angiotensin converting enzyme (ACE) inhibitors (ACEIs) are at increased risk of developing angioedema after rt-PA administration.^        3 ^ In most patients, this side effect presents as a mild and transitory orolingual angioedema.^        3 ^ In general, orolingual angioedema is defined as a localized edematous vascular reaction of either deep dermis or subcutaneous or submucosal tissues caused by dilatation and increased permeability of the capillaries, which pertains to the oral cavity including mouth, lips, and tongue. Following thrombolysis, this unpleasant event has been described in 2% of stroke patients,^        4 ^ However, this prevalence rate has been recently shown in a systematic review to be as high as 17% within the first 4 hours of receiving rt-PA.^   5 ^ The effect of rt-PA on activation of plasminogen into plasmin and the consequent increase in the serum level of bradykinin has been proposed as one probable etiology for orolingual angioedema.^   5 ^ Moreover, it seems that some of the members of the complement system namely C3a, C4a, C5a, and C2-kinin also contribute in the formation of angioedema in patients under rt-PA treatment.^        6 ^ As a clinical experience, we present the two stroke patients using ACEI in whom the orolingual angioedema was developed after rt-PA and even complicated to respiratory distress in one of them.

***Case I***

A 55-year-old obese woman with the previous history of hypertension and cerebrovascular accident (3 years ago) was admitted to Firoozgar General Hospital, Tehran, Iran, complaining from acute weakness in the right limbs. The symptom was started in 2 hours before admission with acute onset. In drug history, she was found under treatment with captopril 25 mg due to the history of hypertension for 6 months. On the initial evaluation, her blood pressure was 170/90 mmHg. She was awake and alert with a left gaze preference, dense right hemiplegia with the National Institutes of Health Stroke Scale (NIHSS) of 19. The serum level of C3 and C4 as the components of the complement system was above the normal range. Full laboratory data is shown in table 1. A non-contrast brain computed tomography (CT) scan showed no hemorrhage, but a left dense middle cerebral artery (MCA) sign was seen. Based on neither historical nor laboratory contraindication for thrombolysis, she received a total of 90 mg IV rt-PA approximately 3 hours after symptom onset with 10% of total dose given as a bolus and 90% of it was given within 1 hour. Within minutes after completing the rt-PA infusion, she developed the edematous lip and tongue swelling. She was treated with 0.5 mg subcutaneous epinephrine and 200 mg hydrocortisone (100 mg twice per day for 48 hours). After 30 minutes, the improvement of her orolingual angioedema was started and completely resolved within 36 hours. However, the neurologic deficits the improved dramatically resulting in an NIHSS of 10 in the 2^nd^ day. A repeated brain CT scan at 24 hours post rt-PA infusion showed an improvement with changes of infarction restricted to the superior division of the left MCA.

***Case II***

A 63-year-old man with a history of hypertension was referred to the emergency ward of the Firoozgar General Hospital presenting with acute left side weakness. The patient was under treatment with captopril for 6 months and his blood pressure was measured as 140/80 mmHg on the initial evaluation. He was alert with dense left hemiplegia and his NIHSS was 13. Elevated serum level of both C3 and C4 was detected in laboratory investigation (Table 1). A non-contrast brain CT scan showed neither hemorrhage, nor early ischemic changes, and nor hyperdense vessel signs. In total, he received 54 mg IV rt-PA with 10% given as a bolus approximately 3.5 hours after symptom onset. Thirty minutes after completing the rt-PA infusion, he developed the left lower lip and tongue swelling (Figure 1). Physical examination revealed an edematous tongue and asymmetric edema in the left lower lip with increased respiratory distress. He was treated with hydrocortisone and epinephrine; however, we had to intubate the patient in order to decrease his respiratory distress. The next day, he was extubated and the repeated brain CT scan also revealed an infarction in the right MCA territory. The patient improved neurologically to an NIHSS of 11 on the discharge day.

**Table 1:** Laboratory data in two reported cases

**Test**	**Value**	**Normal ** **range**
C3 (mg/dl)	Case I: 198 Case II: 212	90-180
C4 (mg/dl)	Case I: 66 Case II: 74	10-40
WBC (total cells/mcl)	Case I: 8600 Case II: 11200	3500-10500
PMN (%)	Case I: 62 Case II: 59	40-80
Eosinophils (%)	Case I: 4 Case II: 6	1-6
Lymphocytes (%)	Case I: 33 Case II: 33	20-40

WBC: White blood cells; PMN: Polymorphonuclear

Though mild and spontaneously reversible in most patients,^        3 ^ orolingual angioedema can become a life-threatening complication of alteplase therapy in stroke patients.^        7 ^

**Figure 1:**
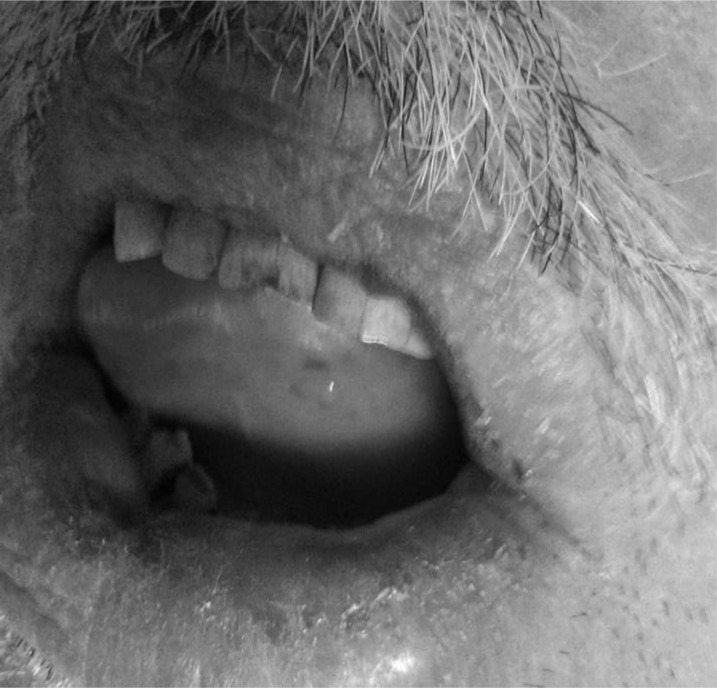
Orolingual angioedema (left lower lip and tongue swelling) after recombinant tissue plasminogen activator infusion in a stroke patient under treatment with captopril

The risk of this unexpected complication is increased to higher rates among those under ACEI medication. The underlying mechanism by which angioedema is produced by rt-PA is explained through the increased level of bradykinin resulting from the cleavage of high-molecular-weight kininogen.^   8 ^ This increased bradykinin has vasodilatory properties, increases vascular permeability, and might be induce angioedema.^        6 ^ While the ACE normally inactivates bradykinin, the use of ACEIs accumulates bradykinin level that consequently makes stroke patients more susceptible to develop the angioedema following rt-PA infusion.^        9 ^ Of note, elevated serum level of the components of complement system (C3 and C4) was observed in both reported cases in our study, which could be in favor of the involvement of complement system in the etiology of angioedema in these patients.

In our report, both patients were consuming captopril to control their hypertension. The orolingual angioedema was rapidly progressive during the 1^st^ minutes after the infusion of rt-PA. Similar to previous reports,^3^^,^^8^ angioedema was asymmetric in our cases and was occurred in the same side as their ipsilateral hemiparesia. It might be related to the infarction of the contralateral insular cortex, which promotes the autonomic dysfunction and vasomotor changes in the hemiparetic side.^8^^,^^10^ 

We administered the epinephrine and corticosteroid to manage the angioedema. However, the severity of respiratory distress necessitated intubation in one of the patients. This intervention is not required the most often because the course of orolingual angioedema is benign and self-limited in the majority of patients.^2^^,^^3^^,^^11^ In conclusion, orolingual angioedema must be taken into account as one probable post rt-PA complication in stroke patients who are under treatment with ACE inhibitors. Although they often answered to epinephrine and corticosteroid, the physicians should be aware of respiratory distress in severe cases in which the airway management is necessary. We hereby presented the first two reported Iranian stroke patients who developed orolingual angioedema following rt-PA therapy. Regarding the increasing trend of rt-PA administration in Iran,^   12 ^ it seems necessary to pay more attention toward orolingual angioedema as a potential adverse event that can become even a life-threatening complication in stroke patients, especially among those under ACEIs medication. Therefore, patients may require the urgent lifesaving procedures such as intubation or tracheotomy.

## Notes:

**How to cite this article: **Motamed MR, Nasiri F, Fereshtehnejad SM, Mehrpour M, Zamani B, Haghi-Ashtiani B, et al. Complicated orolingual angioedema after recombinant tissue plasminogen activator treatment in stroke patients under angiotensin converting enzyme inhibitor: Report of two cases. Iran J Neurol 2015; 14(4): 225-7.
